# Risk factors for adjacent segment degeneration after posterior lumbar fusion surgery in treatment for degenerative lumbar disorders: a meta-analysis

**DOI:** 10.1186/s13018-020-02032-7

**Published:** 2020-12-03

**Authors:** Tao Wang, Wenyuan Ding

**Affiliations:** 1 Wuxi No.9 People Hospital, Wuxi, China; 2 Department of Spinal Surgery, The Third Hospital of Hebei Medical University, No. 139 Ziqiang Road, Shijiazhuang, 050051 China

**Keywords:** Incidence, Risk factors, Adjacent segment degeneration, Lumbar, Fusion surgery, Posterior, Meta-analysis

## Abstract

**Study design:**

A meta-analysis.

**Objective:**

We performed a meta-analysis to explore the incidence and risk factors of adjacent segment degeneration (ASD) after posterior lumbar fusion surgery.

**Methods:**

An extensive search of the literature was performed in English database of PubMed, Embase, and Cochrane Library, and Chinese database of CNKI and WANFANG (up to May 2020). We collected factors including demographic data, surgical factor, and sagittal parameters. Data analysis was conducted with RevMan 5.3 and STATA 12.0.

**Results:**

Finally, 19 studies were included in the final analysis. In our study, the rate of ASD after posterior lumbar fusion surgery was 18.6% (540 of 2896). Our data also showed that mean age, body mass index (BMI), the history of smoking and hypertension, preoperative adjacent disc degeneration, long-segment fusion, preoperative superior facet violation, high lumbosacral joint angle, pre- and post-operative L1-S1 sagittal vertical axis (SVA), post-operative lumbar lordosis (LL), and preoperative pelvic incidence (PI) were associated with the development of ASD. However, gender, history of diabetes, bone mineral density (BMD), preoperative Oswestry Disability Index (ODI) and Japanese Orthopedic Association (JOA), the type of fusion (PLIF vs TLIF), type of bone graft (auto- vs allograft), fusion to S1(vs non-fusion to S1), diagnose (lumbar disc herniation, lumbar spinal stenosis, lumbar spondylolisthesis), preoperative pelvic tilt (PT), LL and sacral slope (SS), post-operative SS, PT and PI were not associated with the development of ASD.

**Conclusions:**

In our study, many factors were correlated with the risk of ASD after posterior lumbar fusion surgery. We hope this article can provide a reference for spinal surgeons in treatment for lumbar degenerative diseases.

**Supplementary Information:**

**Supplementary information** accompanies this paper at 10.1186/s13018-020-02032-7.

## Introduction

Due to good clinical results, posterior lumbar fusion surgeries have been widely used in treatment for various lumbar degenerative diseases. Though the initially good clinical results after fusion, biomechanical change of the spine caused by fusion may accelerate the degeneration of the adjacent segment [1]. So, adjacent segment disease or adjacent segment degeneration (ASD) is considered to be a potential long-term complication of spinal fusion. The rate of ASD, considered radiographic changes without symptom, ranges from 5.2 to 49% in various studies after posterior lumbar fusion surgery [2].

Some researchers found that ASD may be caused by lumbar fusion, which can induce abnormal intradiscal pressure and too much movement at the adjacent spinal levels, resulting in abnormal discal stress distribution [3, 4]. However, Battie et al. [5] found that ASD after fusion was a natural process that was not related to fusion surgery. Recent articles have reported the risk factors for ASD including older age, female, expression of the estrogen receptor, the number of instrumented level, pre-existing degenerative condition at an adjacent motion segment, sagittal alignment change [6–10]. As far as we know, the risk factors for ASD remain controversial. Therefore, this study aims to explore the incidence and risk factors of ASD following posterior lumbar fusion surgery for degenerative lumbar disorders.

## Methods

### Search strategy

We searched for the English and Chinese language studies with the keywords: “adjacent segment degeneration” or “ASD”, and “lumbar surgery” in English database of PubMed, Embase, and Cochrane Library and Chinese database of CNKI and WANFANG. There was no limitation on the date of publication, which covered all previously published studies up to May 2020.

### Eligibility criteria

Included articles must satisfy: (1) study population must be adult patients; (2) measured outcomes of the incidence and risk factors of ASD; (3) comparison: ASD group and non-ASD group; (4) the study must be meet the definition of ASD (defined as a radiological change in which narrowing of disc height was ≥3 mm, the progressive slipping of adjacent segments was ≥3 mm (in comparison with preoperative flexion and extension lateral radiographs), and the posterior opening of adjacent segments was 5°; (5) follow-up of more than 2 years; and (6) patients with lumbar disc herniation, lumbar spinal stenosis, lumbar spondylolisthesis. Studies were excluded if they (1) were abstracts, letters, reviews, or case reports; (2) had repeated data; (3) did not report outcomes of interest;(4) patients treated for lumbar trauma, tumor, infection, inflammation, scoliosis; and (5) patients underwent any other lumbar surgery.

### Data extraction and outcome measures

The data included the general characteristics of each study and the outcomes measured. General characteristics included first author, year of publication, country, the number of ASD patients and total patients, follow-up time, type of article, shown in Table 1. The outcomes include the rate of every risk factors. When the same population was reported in several publications, we retained only the most informative article or complete work to avoid duplication of information. Data were extracted independently by two authors. Any disagreements concerning paper eligibility were resolved by discussion and consensus. Test for risk of publication bias. We performed a visual inspection of the funnel plot for publication bias. The funnel plot should be asymmetric when there is publication bias and symmetric in the case of no publication bias. We performed Egger and Begg tests to measure the funnel plot asymmetry using a significance level of *p*<0.10. The trim and fill computation was used to estimate the effect of publication bias. Sensitive analysis overall because of the low heterogeneity of every factor, so we do not calculate sensitive analysis.

**Table 1:** Characteristics of included studies

First author	Year	Country	No. of participants		Follow up time (years)	Study type
			ASD	Total		
Guoquan Zheng [12]	2020	China	17	200	7	Retrospective
HUANG LIN [13]	2017	China	40	221	2-4	Retrospective
HUANG MI [14]	2014	China	18	109	2	Retrospective
Hui Wang [15]	2017	China	15	237	4-6	Retrospective
Jinqian Liang [16]	2014	China	28	84	5	Retrospective
Takahiro Makino [17]	2018	Japan	5	41	2	Retrospective
Yeon Heo [18]	2015	Korea	33	378	6	Retrospective
Zhao-Ming Zhong [19]	2017	China	18	154	5	Retrospective
Zhaoxin Ma [20]	2019	China	22	71	3-6	Retrospective
Shuta Ushio [21]	2019	Japan	22	50	2-9	Retrospective
Seyed Reza Bagheri [22]	2019	Iran	76	630	7-11	Retrospective
Jun Seok Bae [23]	2010	Korea	11	103	6-9	Retrospective
Kyoung-Suok Cho [24]	2009	Korea	9	81	8	Retrospective
Jaewan Soh [25]	2013	Korea	21	55	5	Retrospective
Jigar Anandjiwala [26]	2011	Korea	14	68	5	Retrospective
Masayuki Miyagi [27]	2013	Japan	14	23	4	Retrospective
Bai-Ling Chen [28]	2011	China	11	49	2-4	Retrospective
LI WEISHI [29]	2018	China	50	72	6	Retrospective
GUO YANG [30]	2020	China	116	270	3-5	Retrospective

### Statistical analysis

Only dichotomous outcomes were mentioned in our study, so odd ratios (OR) and 95% confidence intervals (CI) were calculated for outcomes. A *p* value<0.05 was judged as statistically significant. Random-effects or fixed-effects models were used depending on the heterogeneity of the studies included. Heterogeneity was analyzed with both the chi-squared test *I*^2^ test, where *p* value of<0.10 for the chi-squared and *I*^2^>50% implied heterogeneity [11]. All statistical analyses were performed using Review Manager version 5.3 (The Cochrane Collaboration, Oxford, UK) and STATA 12.0 (Stata Corporation, College Station, TX, USA).

## Results

### Study identification and selection

**Fig. 1:**
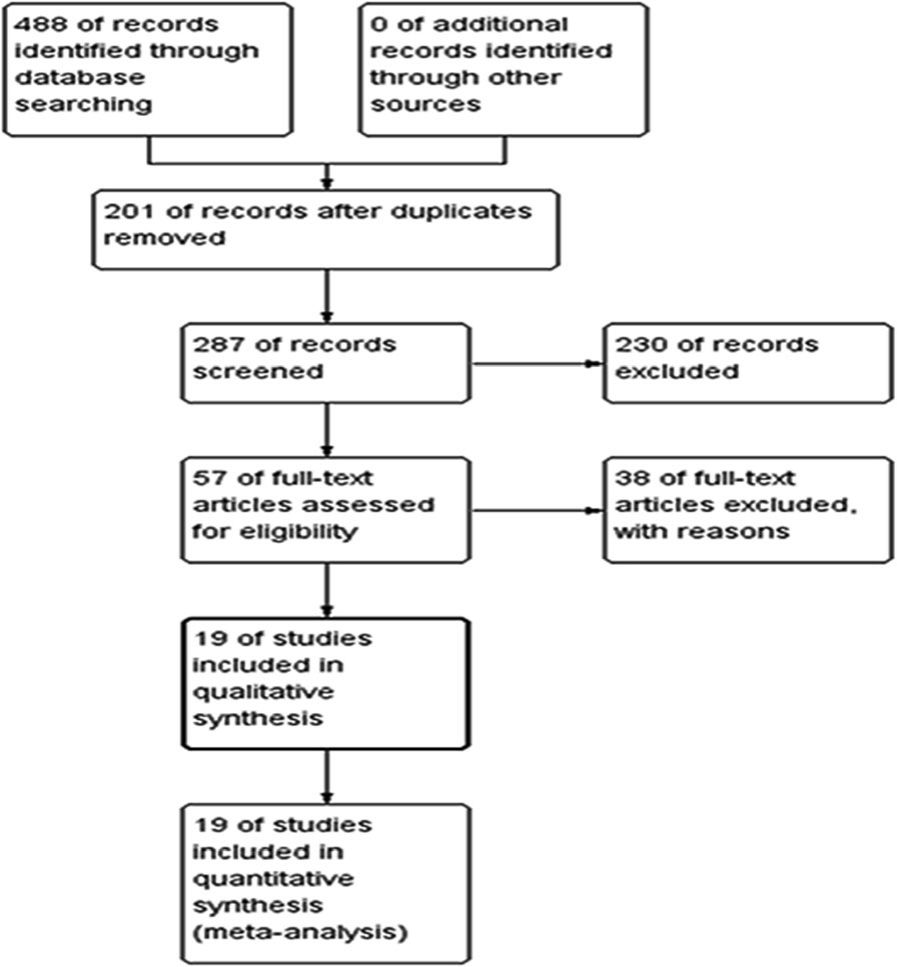
Flow diagram of the study selection

Initially, we collected a total of 488 records by the database search. A total of 201 records were excluded due to repetition and 230 records were removed for review based on the titles and abstracts. The remaining 57 records were retrieved for inclusion criteria and 28 of them were excluded, 10 did not report outcomes of interest. Finally, 19 articles that met our inclusion criteria were included in the present meta-analysis. The selection process included in this meta-analysis is shown in Fig. 1.

### Baseline characteristics and quality assessment

The main characteristics of the 19 articles (from 23 to 630 patients) that were published before May 2020 included in the meta-analysis are presented in Table 1. A total of 540 patients were suffering from ASD after posterior lumbar fusion surgery in a total of 2896 patients. According to the 19 included studies, the rate of ASD was 18.6% (ranged from 8.5 to 69.4%).

Because all studies included were retrospective studies, we used the Newcastle Ottawa Quality Assessment Scale (NOQAS) to assess the quality of each study. This scale for non-randomized case-controlled studies and cohort studies was used to allocate a maximum of nine points for the quality of selection, comparability, exposure, and outcomes for study participants. Of these studies, 13 studies scored eight points and 6 studies scored seven points. Hence, the quality of each study was relatively high (Table 2).

**Table 2:** The quality assessment according to the Newcastle Ottawa Quality Assessment Scale (NOQAS) of each study

Study	Selection	Comparability	Exposure	Total score
Guoquan Zheng [12]	3	3	2	8
Huang Lin [13]	3	3	2	8
Huang Mi [14]	3	2	3	8
Hui Wang [15]	2	3	3	8
Jinqian Liang [16]	2	3	3	8
Takahiro Makino [17]	3	3	2	8
Yeon Heo [18]	2	2	3	7
Zhao-Ming Zhong [19]	3	2	2	7
Zhaoxin Ma [20]	3	2	3	8
Shuta Ushio [21]	2	2	3	7
Seyed Reza Bagheri [22]	3	2	3	8
Jun Seok Bae [23]	3	2	2	7
Kyoung-Suok Cho [24]	3	2	3	8
Jaewan Soh [25]	2	2	3	7
Jigar Anandjiwala [26]	3	2	3	8
Masayuki Miyagi [27]	3	2	3	8
Bai-Ling Chen [28]	3	2	3	8
Li WeishI [29]	2	2	3	7
Guo Yang [30]	2	3	3	8

### Assessment of risk factors of ASD

#### Age

**Fig. 2:**
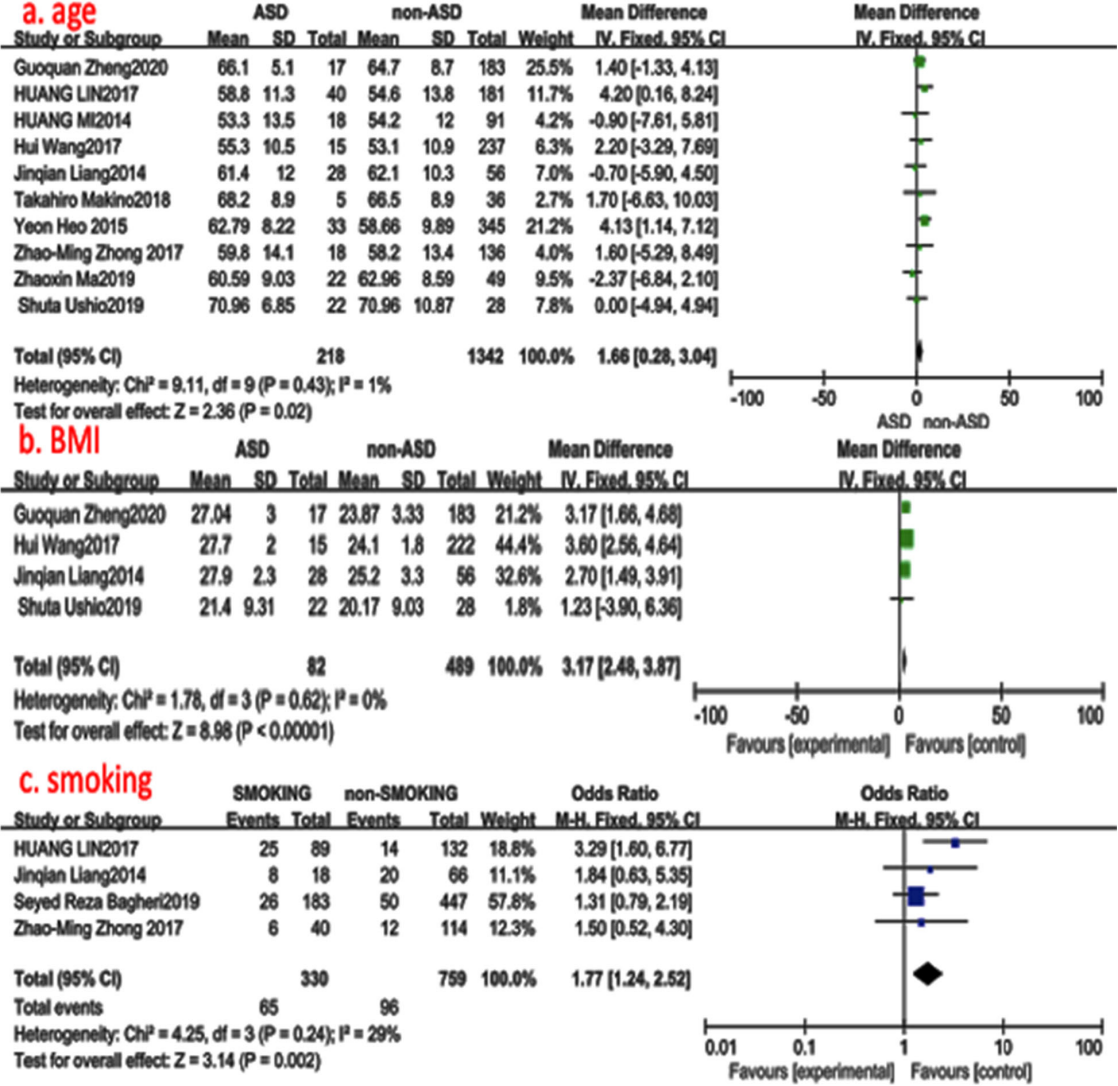
**a** The standardized mean difference (SMD) estimate for preoperative age in 2 groups. **b** The standardized mean difference (SMD) estimate for preoperative body mass index in 2 groups. **c** The odds ratio (OR) estimate for the history of smoking. CI = confidence interval, df = degrees of freedom, M-H = Mantel–Haenszel

Ten studies (1560 of 2896 patients) [12–21] reported the age of patients at an operational time between ASD group and non-ASD group. There was no significance in the test for heterogeneity and the studies had low heterogeneity (*p* for heterogeneity = 0.43; *I*^2^ = 1%, Fig. 2). The meta-analysis showed that age was associated with a significant increase in the incidence of ASD (fixed-effects model; *p* = 0.02, SMD = 1.66, 95% CI [0.28, 3.04], Fig. 2).

#### Body mass index (BMI)

Four studies (571 of 2896 patients) [12, 15, 16, 21] reported BMI of patients at an operational time between ASD group and non-ASD group. There was no significance in the test for heterogeneity and the studies had low heterogeneity (*p* for heterogeneity = 0.62; *I*^2^ = 0%, Fig. 2). The meta-analysis showed that BMI was associated with a significant increase in the incidence of ASD (fixed-effects model; *p*<0.0001, SMD = 3.17, 95% CI [2.48, 3.87], Fig. 2).

#### History of smoking

Four studies (1250 of 2896 patients) [13, 16, 19, 22] reported a history of smoking between ASD group and non-ASD group. There was no significance in the test for heterogeneity and the studies had low heterogeneity (*p* for heterogeneity = 0.24; *I*^2^ = 29%, Fig. 2). The meta-analysis showed that the history of smoking was associated with a significant increase in the incidence of ASD (fixed-effects model; *p* = 0.0002, OR = 1.77, 95% CI [1.24, 2.52], Fig. 2).

#### Gender

**Fig. 3:**
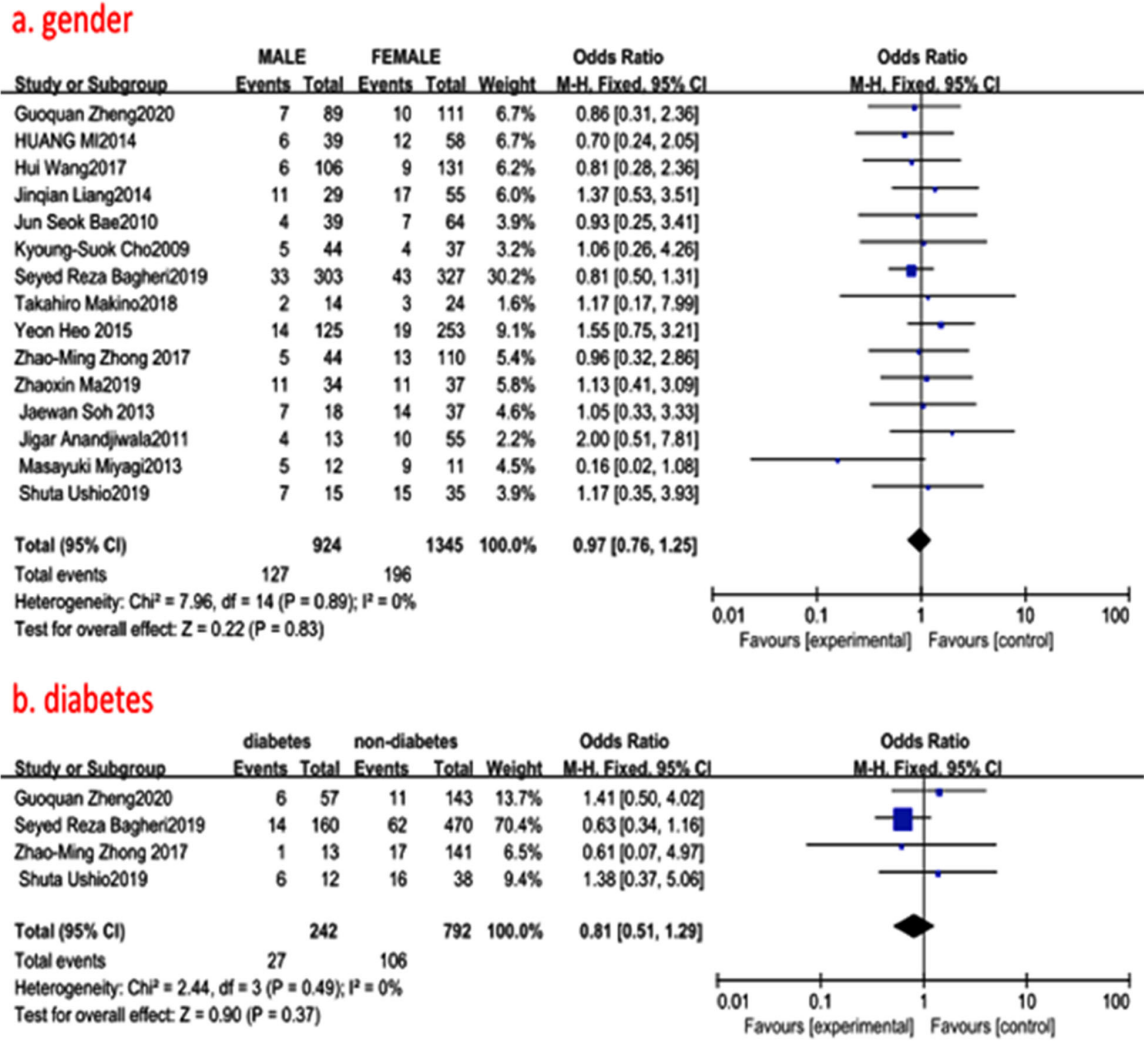
**a** The odds ratio (OR) estimate for gender. **b** The odds ratio (OR) estimate for the history of diabetes. CI = confidence interval, df = degrees of freedom, M-H=Mantel–Haenszel

Fifteen studies (2592 of 2896 patients) [12, 14–27] reported gender between ASD group and non-ASD group. There was no significance in the test for heterogeneity and the studies had low heterogeneity (*p* for heterogeneity = 0.89; *I*^2^ = 0%, Fig. 3). The meta-analysis showed that gender was not associated with a significant increase in the incidence of ASD (fixed-effects model; *p* = 0.83, OR = 0.97, 95% CI [0.76, 1.25], Fig. 3).

#### History of diabetes

Four studies (1167 of 2896 patients) [12, 19, 21, 22] reported a history of diabetes between ASD group and non-ASD group. There was no significance in the test for heterogeneity and the studies had low heterogeneity (*p* for heterogeneity = 0.49; *I*^2^ = 0%, Fig. 3). The meta-analysis showed that the history of diabetes was not associated with a significant increase in the incidence of ASD (fixed-effects model; *p* = 0.37, OR = 0.81, 95% CI [0.51, 1.29], Fig. 3).

#### Bone mineral density (BMD)

**Fig. 4:**
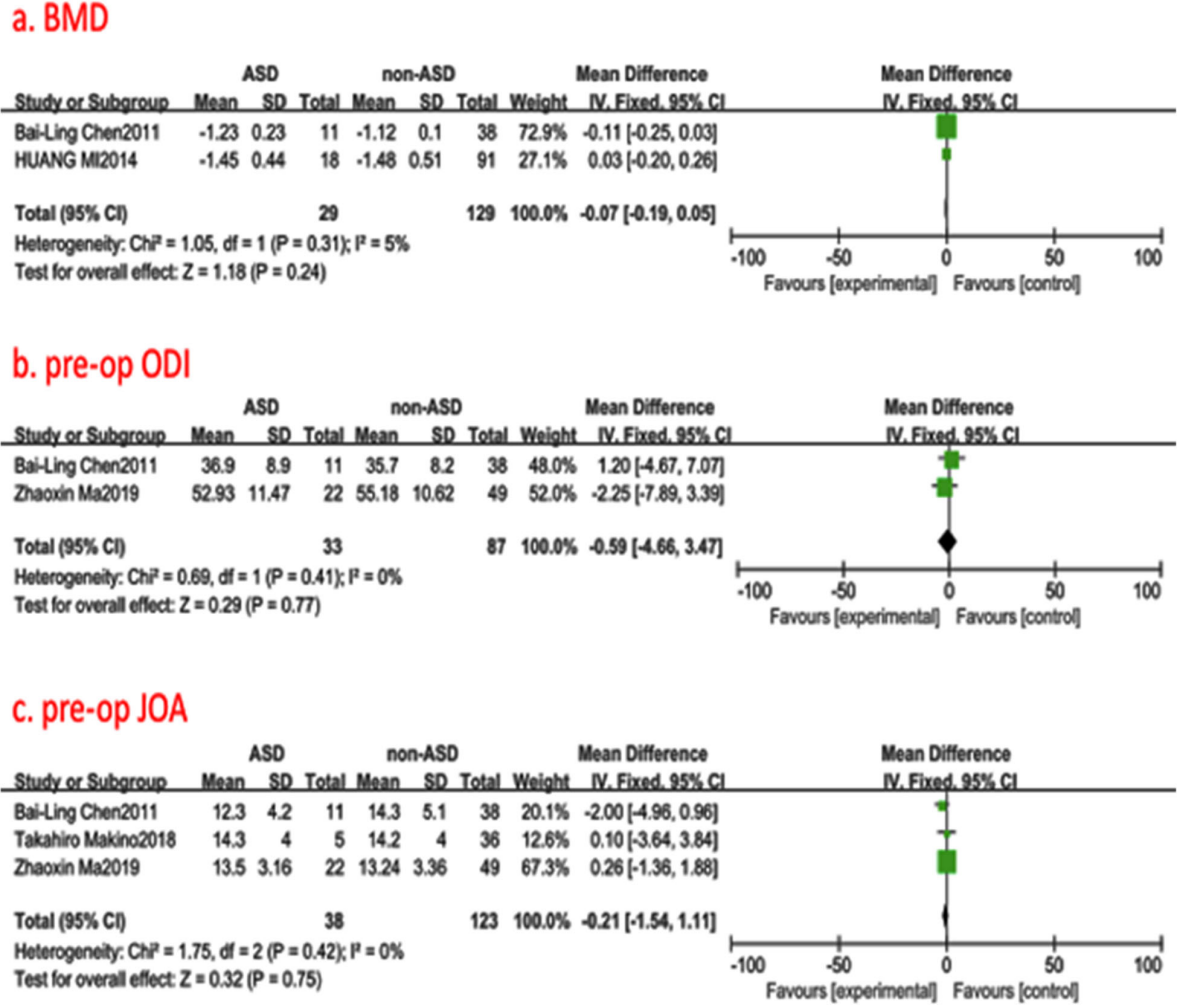
**a** The standardized mean difference (SMD) estimate for bone mineral density in 2 groups. **b** The standardized mean difference (SMD) estimate for preoperative ODI score in 2 groups. **c** The standardized mean difference (SMD) estimate for preoperative JOA score in 2 groups. df = degrees of freedom, ODI = Oswestry disability index. JOA = Japanese Orthopedic Association, M-H = Mantel–Haenszel

Two studies (158 of 2896 patients) [14, 28] reported BMD at the operational time between ASD group and non-ASD group. There was not significant in the test for heterogeneity and the studies had low heterogeneity (*p* for heterogeneity = 0.31; *I*^2^ = 5%, Fig. 4). The meta-analysis showed that BMD was not associated with a significant increase in the incidence of ASD (fixed-effects model; *p* = 0.24, SMD = −0.07, 95% CI [−0.19, 0.05], Fig. 4).

#### Preoperative Oswestry Disability Index (ODI)

Two studies (120 of 2896 patients )[20, 28] reported preoperative ODI between ASD group and non-ASD group. There was no significance in the test for heterogeneity and the studies had low heterogeneity (*p* for heterogeneity = 0.41; *I*^2^ = 0%, Fig. 4). The meta-analysis showed that preoperative ODI was not associated with a significant increase in the incidence of ASD (fixed-effects model; *p* = 0.77, SMD = −0.59, 95% CI [−4.66, 3.47], Fig. 4).

#### Preoperative Japanese Orthopedic Association (JOA)

Three studies (161 of 2896 patients) [17, 20, 28] reported preoperative JOA between ASD group and non-ASD group. There was no significance in the test for heterogeneity and the studies had low heterogeneity (*p* for heterogeneity = 0.42; *I*^2^ = 0%, Fig. 4). The meta-analysis showed that preoperative JOA was not associated with a significant increase in the incidence of ASD (fixed-effects model; *p* = 0.75, SMD = −0.21, 95% CI [−1.54, 1.11], Fig. 4).

#### History of hypertension

**Fig. 5:**
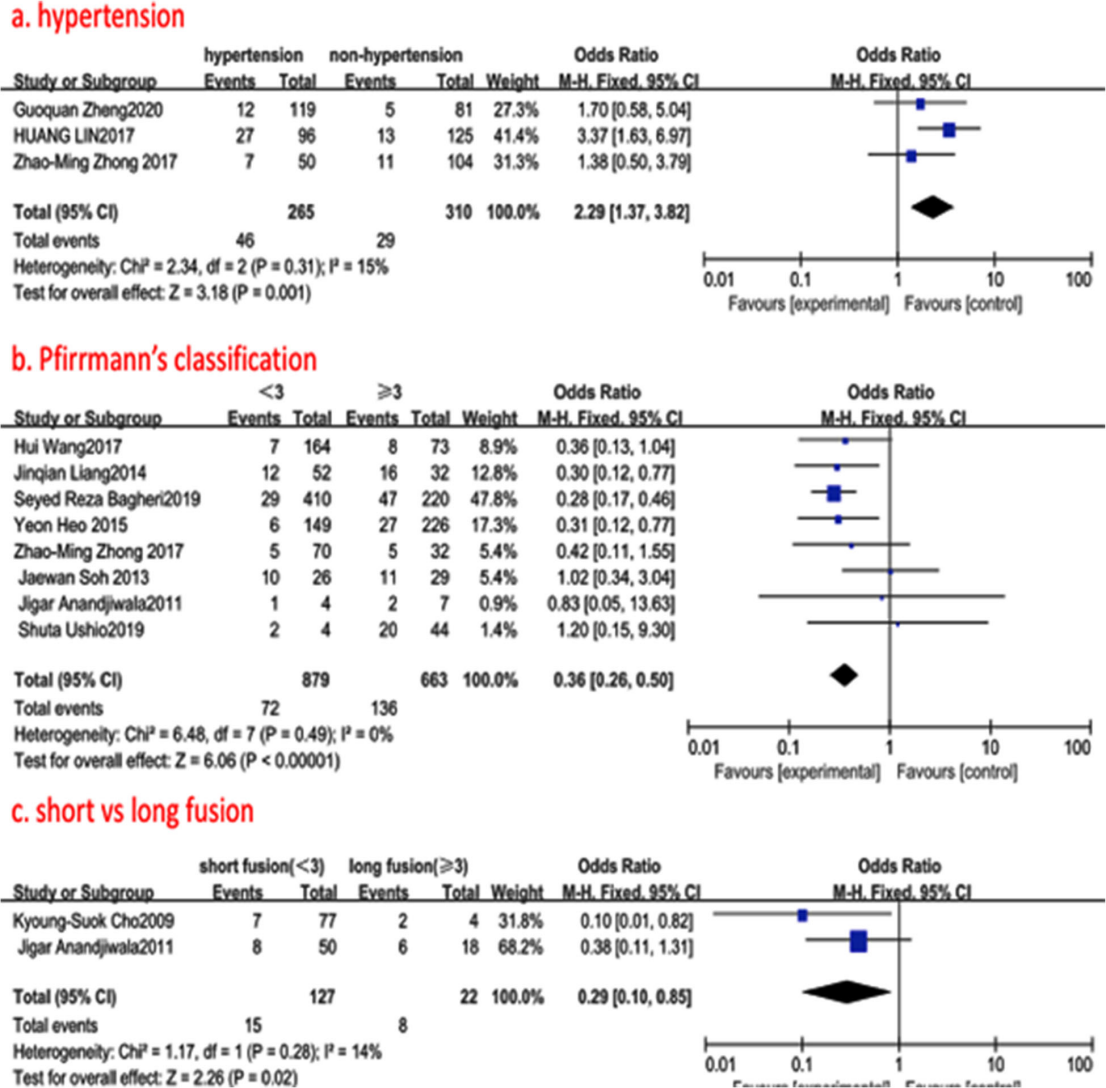
**a** The odds ratio (OR) estimate for the history of hypertension. **b** The odds ratio (OR) estimate for preoperative Pfirrmann’s classification. **c** The odds ratio (OR) estimate for the length of fusion (short vs long fusion). CI = confidence interval, df = degrees of freedom, M-H = Mantel–Haenszel

Three studies (650 of 2896 patients) [12, 13, 19] reported a history of hypertension between ASD group and non-ASD group. There was no significance in the test for heterogeneity and the studies had low heterogeneity (*p* for heterogeneity = 0.31; *I*^2^ = 15%, Fig. 5). The meta-analysis showed that the history of hypertension was associated with a significant increase in the incidence of ASD (fixed-effects model; *p* = 0.001, OR = 2.29, 95% CI [1.37, 3.82], Fig. 5).

#### Preoperative Pfirrmann’s classification at the adjacent segment

Eight studies (1750 of 2896 patients) [15, 16, 18, 19, 21, 22, 25, 26] reported Pfirrmann’s classification between ASD group and non-ASD group. There was no significance in the test for heterogeneity and the studies had low heterogeneity (*p* for heterogeneity = 0.49; *I*^2^ = 0%, Fig. 5). The meta-analysis showed that preoperative Pfirrmann’s classification of more than three levels was associated with a significant increase in the incidence of ASD (fixed-effects model; *p*<0.0001, OR = 0.36, 95% CI [0.26, 0.50], Fig. 5).

#### Short versus long fusion

Two studies (172 of 2896 patients) [24, 26] reported the length of fusion between ASD group and non-ASD group. There was no significance in the test for heterogeneity and the studies had low heterogeneity (*p* for heterogeneity = 0.20; *I*^2^ = 14%, Fig. 5). The meta-analysis showed that long fusion ≥3 was associated with a significant increase in the incidence of ASD (fixed-effects model; *p* = 0.02, OR = 0.29, 95% CI [0.10, 0.85], Fig. 5).

#### Preoperative superior facet violation

**Fig. 6:**
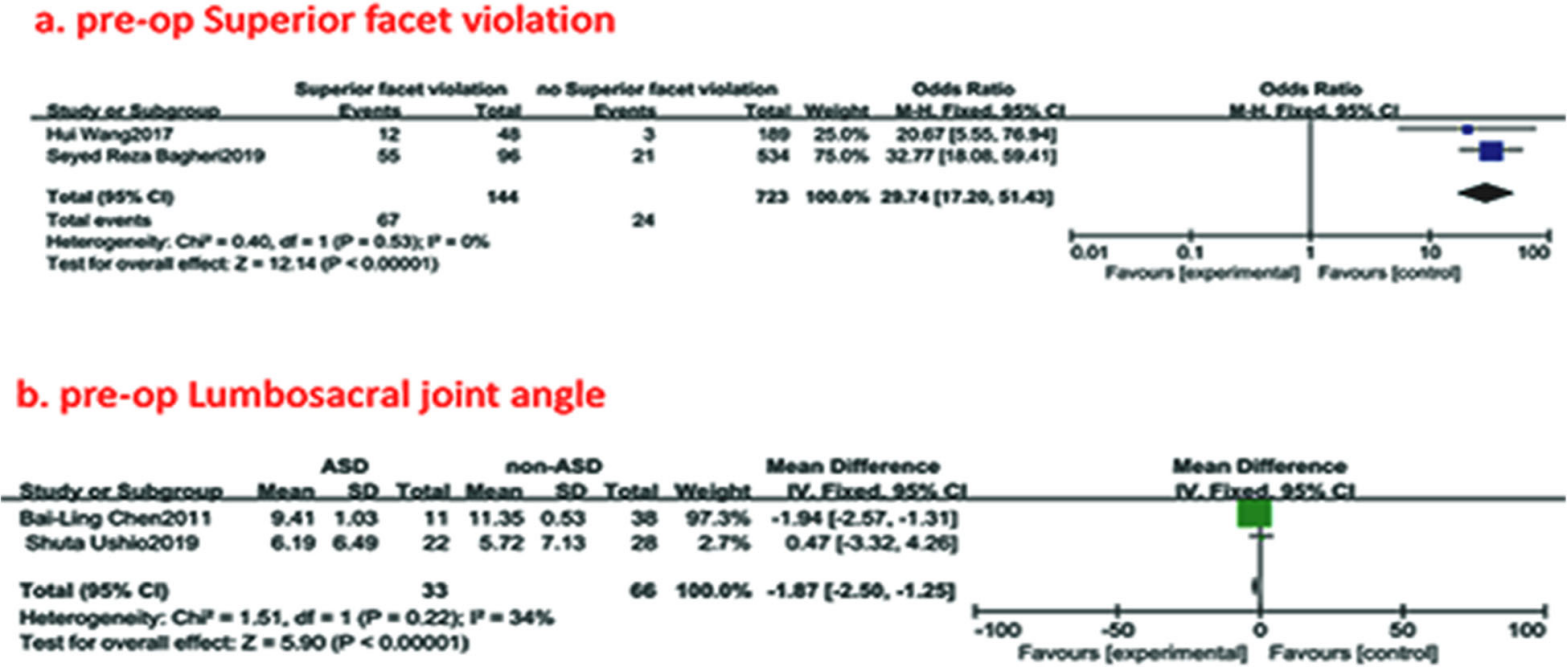
**a** The odds ratio (OR) estimate for preoperative superior facet violation. **b** The standardized mean difference (SMD) estimate for preoperative lumbar-sacral joint angle in 2 groups. CI = confidence interval, df = degrees of freedom, M-H = Mantel–Haenszel

Two studies (958 of 2896 patients) [15, 22] reported a preoperative superior facet violation between ASD group and non-ASD group. There was no significance in the test for heterogeneity and the studies had low heterogeneity (*p* for heterogeneity = 0.53; *I*^2^ = 0%, Fig. 6). The meta-analysis showed that preoperative superior facet violation was associated with a significant increase in the incidence of ASD (fixed-effects model; *p*<0.00001, OR = 29.74, 95% CI [17.20, 51.43], Fig. 6).

#### Preoperative lumbosacral joint angle

Two studies (99 of 2896 patients) [21, 28] reported a preoperative lumbosacral joint angle between ASD group and non-ASD group. There was no significance in the test for heterogeneity and the studies had low heterogeneity (*p* for heterogeneity = 0.22; *I*^2^ = 34%, Fig. 6). The meta-analysis showed that the preoperative lumbosacral joint angle was associated with a significant increase in the incidence of ASD (fixed-effects model; *p*<0.00001, SMD = −1.87, 95% CI [−2.50, −1.25], Fig. 6).

### Type of fusion (PLIF versus TLIF)

**Fig.7:**
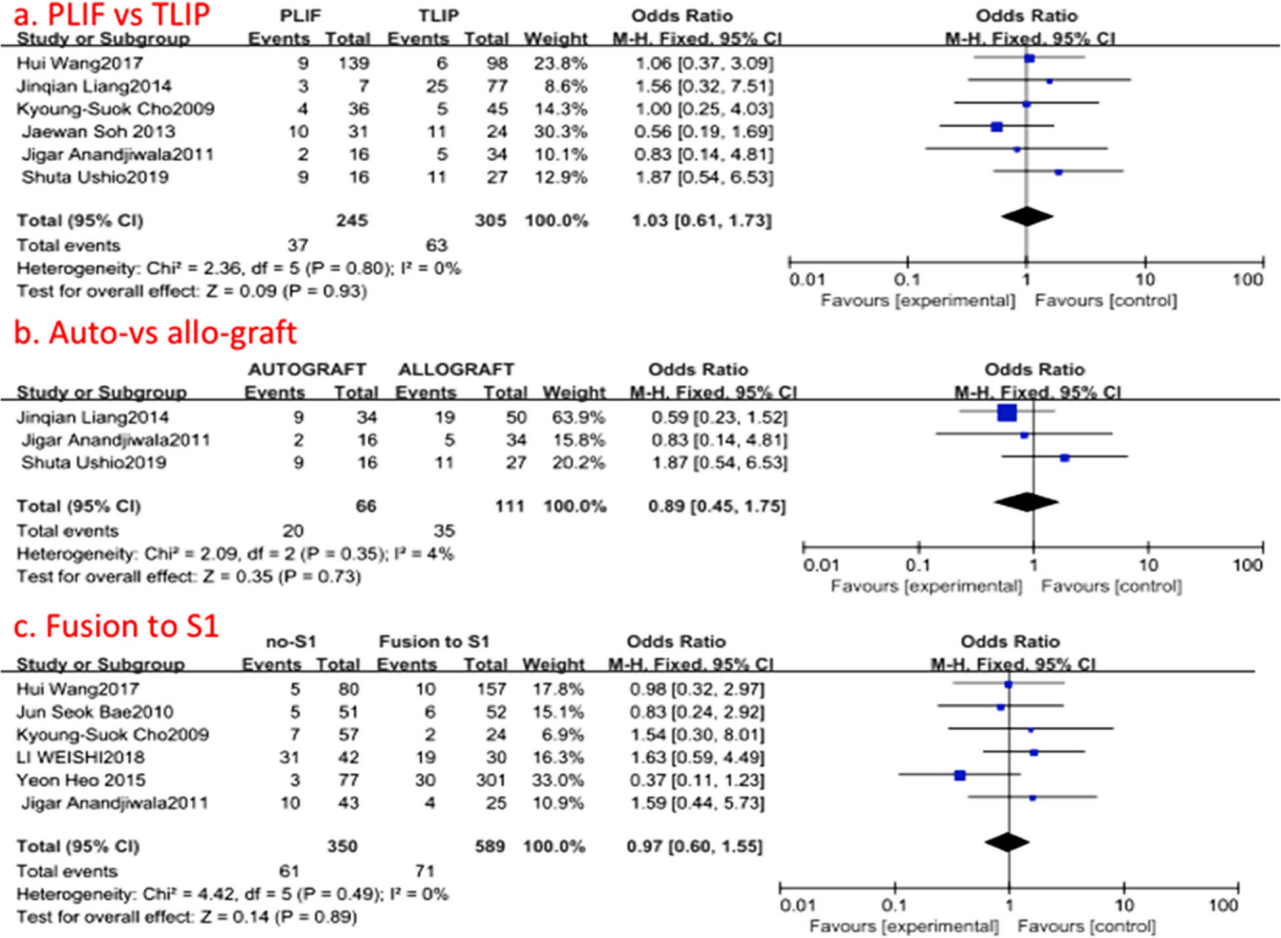
**a** The odds ratio (OR) estimate for the type of fusion (PLIF vs TLIP). **b** The odds ratio (OR) estimate for the type of graft (auto- vs allograft). **c** The odds ratio (OR) estimate for fusion to S1 (vs non-fusion to S1). CI = confidence interval, df = degrees of freedom, M-H = Mantel–Haenszel

Six studies (650 of 2896 patients) [15, 16, 21, 24–26] reported the type of fusion (PLIF versus TLIF) between ASD group and non-ASD group. There was no significance in the test for heterogeneity and the studies had low heterogeneity (*p* for heterogeneity = 0.80; *I*^2^ = 0%, Fig. 7). The meta-analysis showed that type of fusion (PLIF versus TLIF) was not associated with a significant increase in the incidence of ASD (fixed-effects model; *p* = 0.93, OR = 1.03, 95% CI [0.61, 1.73], Fig. 7).

### Type of graft (Auto- versus allograft)

Three studies (232 of 2896 patients) [16, 21, 26] reported the type of graft (auto- versus allograft) between ASD group and non-ASD group. There was no significance in the test for heterogeneity and the studies had low heterogeneity (*p* for heterogeneity = 0.34; *I*^2^ = 4%, Fig. 7). The meta-analysis showed that type of graft (auto- versus allograft) was not associated with a significant increase in the incidence of ASD (fixed-effects model; *p* = 0.73, OR = 0.89, 95% CI [0.45, 1.75], Fig. 7).

#### Fusion to S1 (versus non-fusion to S1)

Six studies (1071 of 2896 patients) [15, 18, 23, 24, 26, 29] reported fusion to S1 between ASD group and non-ASD group. There was no significance in the test for heterogeneity and the studies had low heterogeneity (*p* for heterogeneity = 0.49; *I*^2^ = 0%, Fig. 7). The meta-analysis showed that fusion to S1 was not associated with a significant increase in the incidence of ASD (fixed-effects model; *p* = 0.89, OR = 0.97, 95% CI [0.60, 1.55], Fig. 7).

### Diagnose (lumbar disc herniation, lumbar spinal stenosis, lumbar spondylolisthesis)

**Fig. 8:**
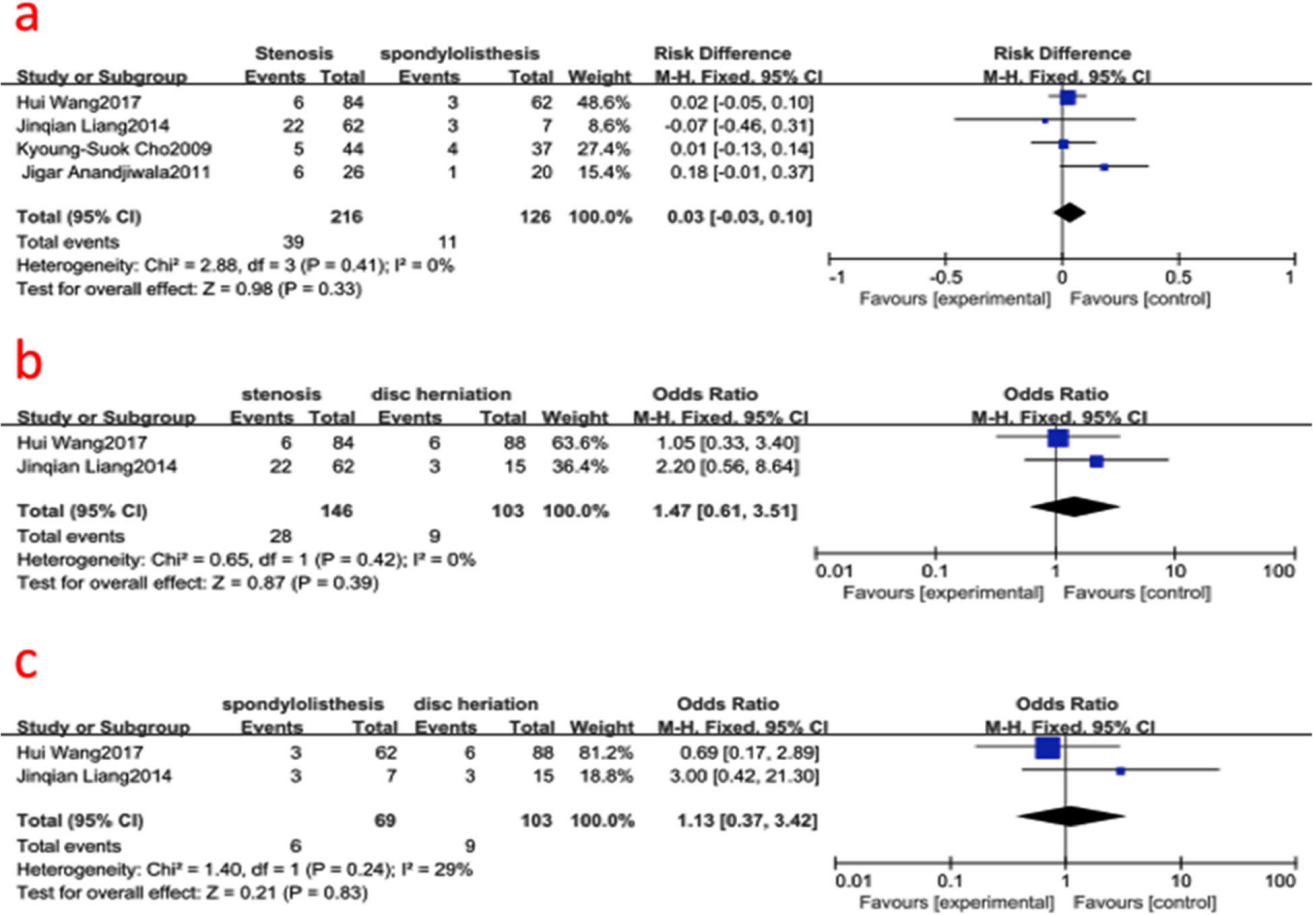
**a** The odds ratio (OR) estimate for diagnosis (lumbar spinal stenosis vs lumbar spondylolisthesis). **b** The odds ratio (OR) estimate for diagnosis (lumbar disc herniation vs lumbar spinal stenosis). **c** The odds ratio (OR) estimate for diagnosis (lumbar disc herniation vs lumbar spondylolisthesis). CI = confidence interval, df = degrees of freedom, M-H = Mantel–Haenszel

Four studies (392 of 2896 patients) [15, 16, 24, 26] reported diagnose (lumbar disc herniation, lumbar spinal stenosis, lumbar spondylolisthesis) between ASD group and non-ASD group. There was no significance in the test for heterogeneity and the studies had low heterogeneity (three *p* for heterogeneity = 0.41, 0.42, 0.24; *I*^2^ = 0, 0, 29%, Fig. 8). The meta-analysis showed that diagnose (lumbar disc herniation, lumbar spinal stenosis, lumbar spondylolisthesis) was not associated with a significant increase in the incidence of ASD (fixed-effects model; *p* = 0.33, 0.39, 0.83; OR = 0.03, 95% CI [−0.03, 0.10]; OR = 1.47, 95% CI [0.61, 3.51]; OR = 1.13, 95% CI [0.37, 3.42], respectively, Fig. 8).

### Pre- and post-operative L1-S1 sagittal vertical axis (SVA)

**Fig. 9:**
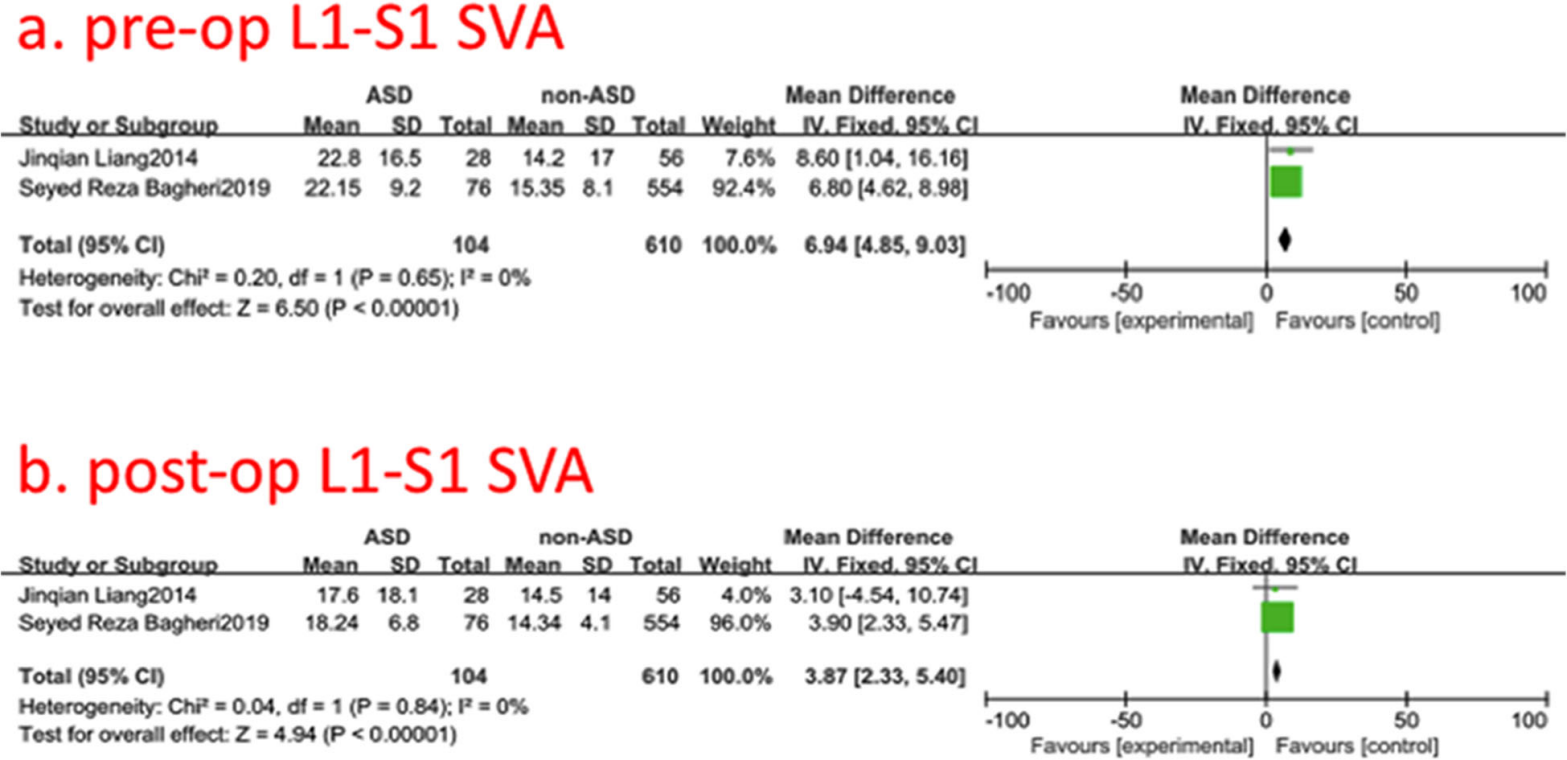
**a** The standardized mean difference (SMD) estimate for preoperative L1-S1 sagittal vertical axis (SVA)in 2 groups. **b** The standardized mean difference (SMD) estimate for post-operative L1-S1SVA in 2 groups. CI = confidence interval, df = degrees of freedom, M-H = Mantel–Haenszel

Two studies (714of 2896 patients) [16, 22] reported pre- and post-operative L1-S1 SVA between ASD group and non-ASD group. There was no significance in the test for heterogeneity and the studies had low heterogeneity (two *p* for heterogeneity = 0.65, 0.84; *I*^2^ = 0, 0%, Fig. 9). The meta-analysis showed that both pre- and post-operative L1-S1 SVA were associated with a significant increase in the incidence of ASD (fixed-effects model; *p*<0.00001, SMD = 6.94, 95% CI [4.85, 9.03]; SMD = 3.87, 95% CI [2.33, 5.40], respectively, Fig. 9).

### Pre- and post-operative pelvic tilt (PT)

**Fig. 10:**
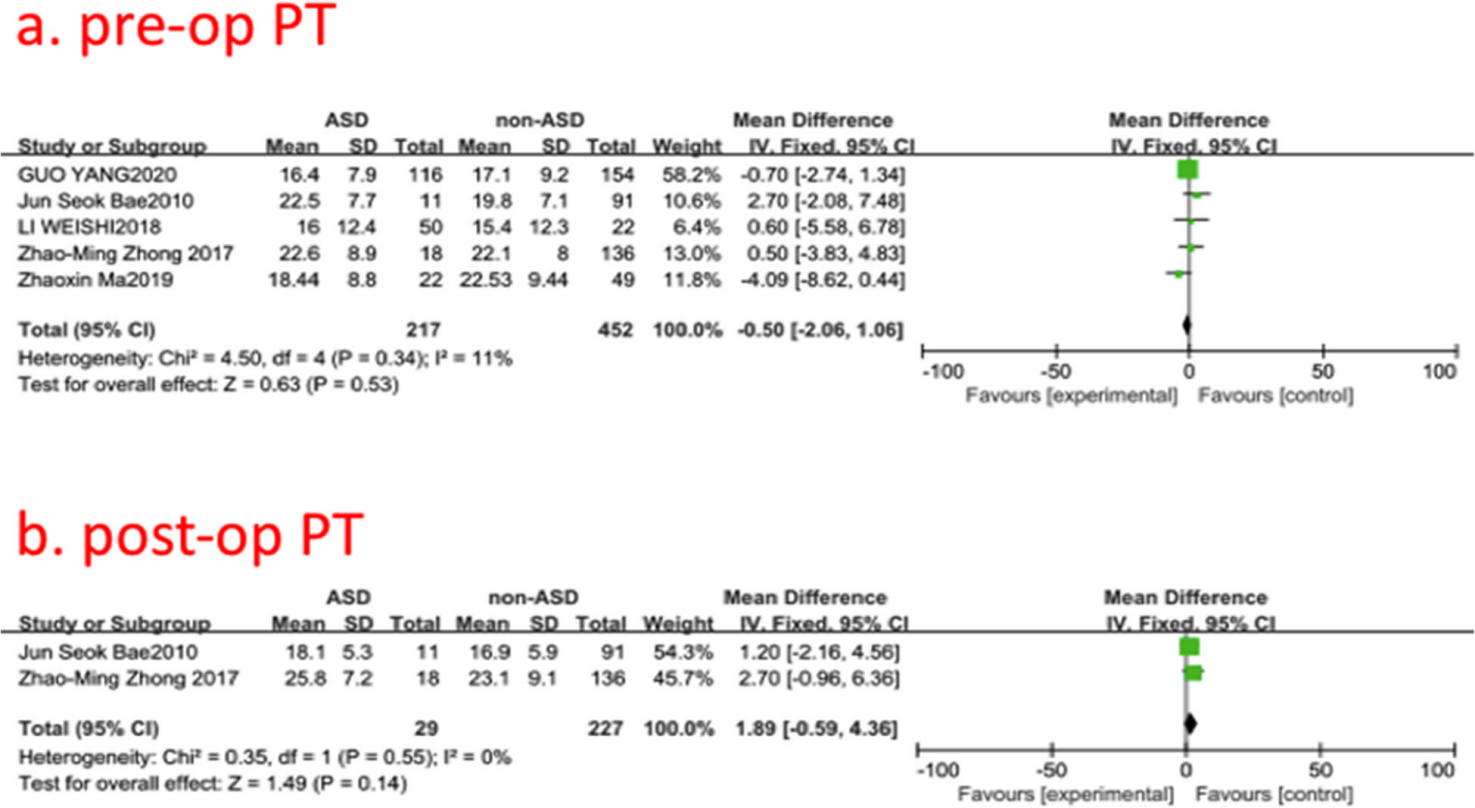
**a** The standardized mean difference (SMD) estimate for preoperative pelvic tilt (PT)in 2 groups. **b** The standardized mean difference (SMD) estimate for post-operative PT in 2 groups. CI = confidence interval, df = degrees of freedom, M-H = Mantel–Haenszel

Five studies (669 of 2896 patients) [19, 20, 22, 29, 30] reported pre- and post-operative PT between ASD group and non-ASD group. There was no significance in the test for heterogeneity and the studies had low heterogeneity (two *p* for heterogeneity = 0.34, 0.55; *I*^2^ = 11, 0%, Fig. 10). The meta-analysis showed that both pre- and post-operative PT were not associated with a significant increase in the incidence of ASD (fixed-effects model; *p* = 0.53, 0.14, SMD = −0.50, 95% CI [−2.06, 1.06]; SMD = 1.89, 95% CI [−0.59,4.36], respectively, Fig. 10).

### Pre- and post-operative sacral slope (SS)

**Fig. 11:**
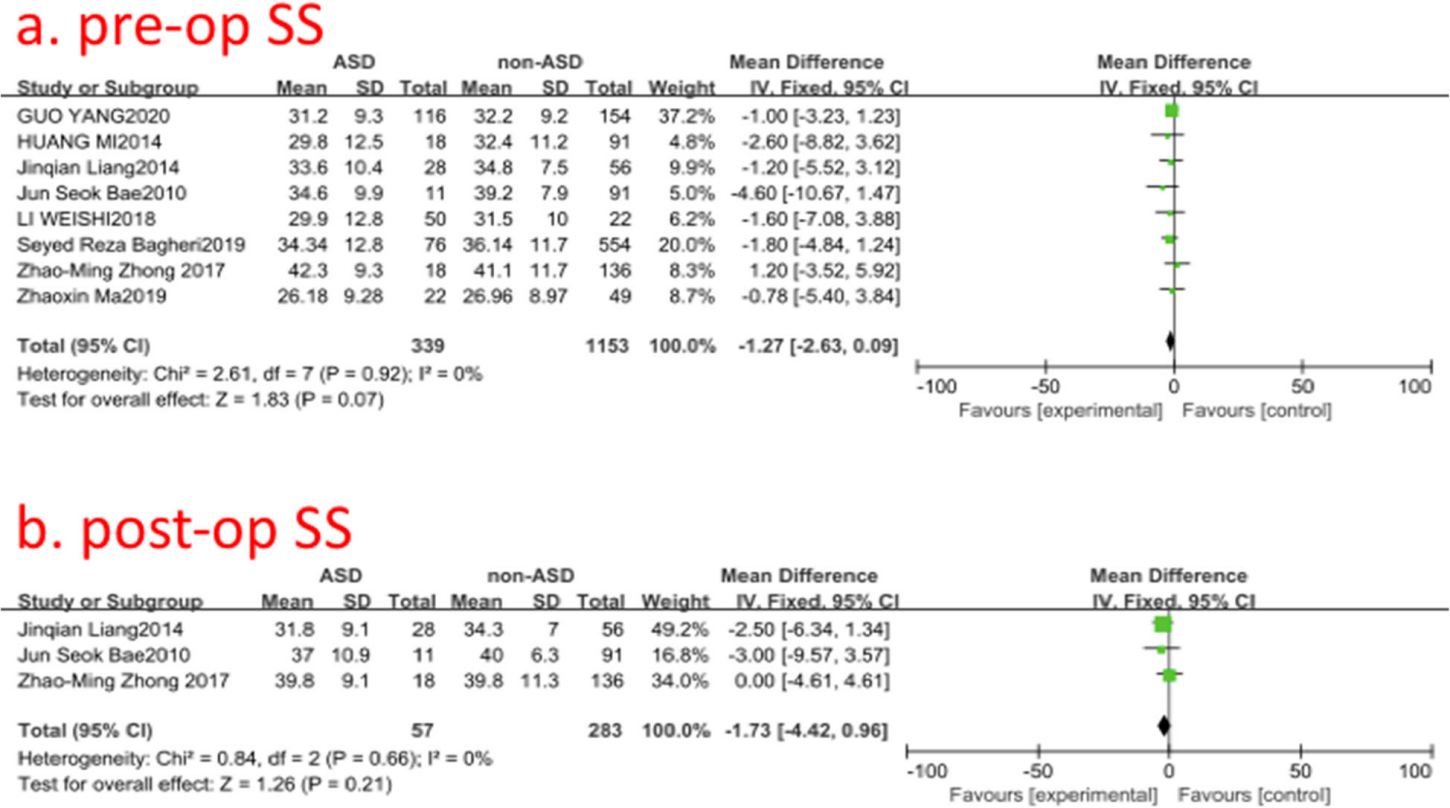
**a** The standardized mean difference (SMD) estimate for preoperative sacral slope (SS) in 2 groups. **b** The standardized mean difference (SMD) estimate for post-operative SS in 2 groups. CI = confidence interval, df = degrees of freedom, M-H = Mantel–Haenszel

Eight studies (1492 of 2896 patients) [14, 16, 19, 20, 22, 23, 29, 30] reported pre- and post-operative SS between ASD group and non-ASD group. There was no significance in the test for heterogeneity and the studies had low heterogeneity (two *p* for heterogeneity = 0.92, 0.66; *I*^2^ = 0, 0%, Fig. 11). The meta-analysis showed that both pre- and post-operative SS was not associated with a significant increase in the incidence of ASD (fixed-effects model; *p* = 0.07, 0.21, SMD = −1.27, 95% CI [−2.63, 0.09]; SMD = −1.73, 95% CI [−4.42, 0.96], respectively, Fig. 11).

### Pre- and post-operative pelvic incidence (PI)

**Fig. 12:**
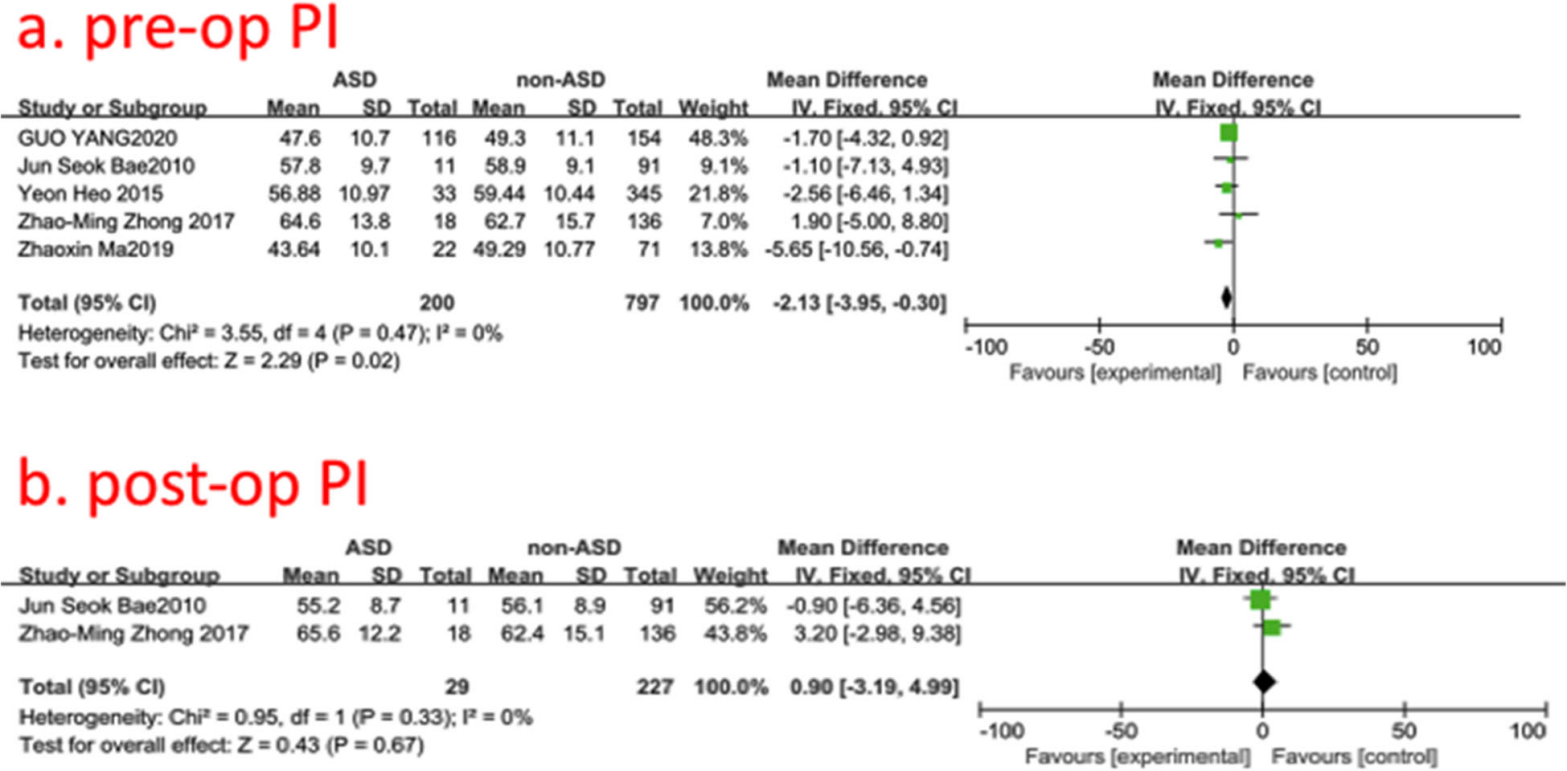
**a** The standardized mean difference (SMD) estimate for preoperative pelvic incidence (PI) in 2 groups. **b** The standardized mean difference (SMD) estimate for post-operative PI in 2 groups. CI = confidence interval, df = degrees of freedom, M-H = Mantel–Haenszel

Five studies (997 of 2896 patients) [18–20, 23, 30] reported pre- and post-operative PI between ASD group and non-ASD group. There was no significance in the test for heterogeneity and the studies had low heterogeneity (two *p* for heterogeneity = 0.47, 0.33; *I*^2^ = 0, 0%, Fig. 12). The meta-analysis showed that preoperative PI was associated with a significant increase in the incidence of ASD; however, post-operative PI was not associated with it (fixed-effects model; *p* = 0.02, 0.67, SMD = −2.13, 95% CI [−3.95, −0.30]; SMD = 0.90, 95% CI [−3.19, 4.99], respectively, Fig. 12).

### Pre- and post-operative lumbar lordosis (LL)

**Fig. 13:**
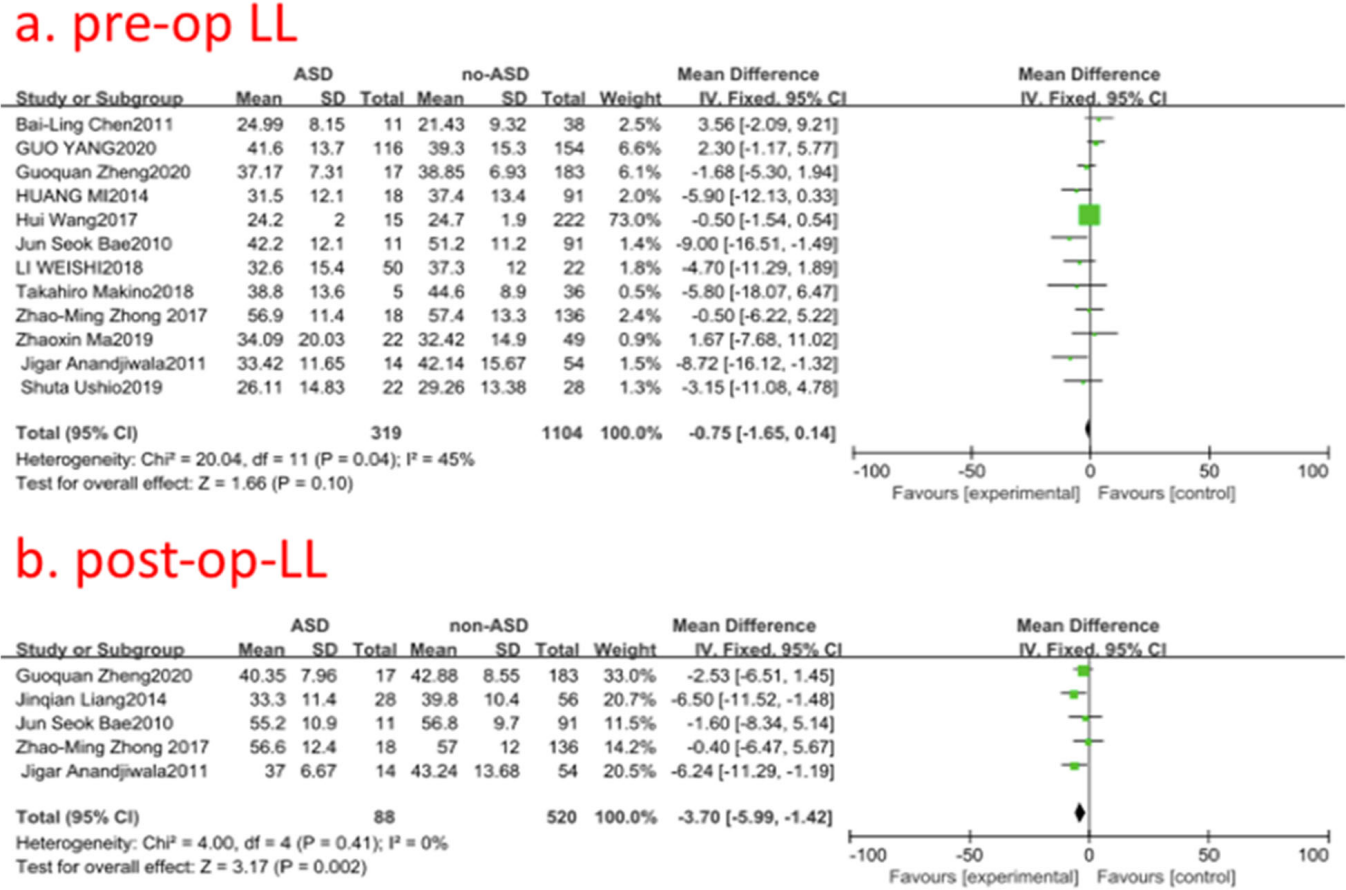
**a** The standardized mean difference (SMD) estimate for preoperative lumbar lordosis (LL) in 2 groups. **b** The standardized mean difference (SMD) estimate for post-operative LL in 2 groups. CI = confidence interval, df = degrees of freedom, M-H = Mantel–Haenszel

Twelve studies (1423 of 2896 patients) [12, 14, 15, 17, 19–21, 23, 26, 28–30] reported pre- and post-operative LL between ASD group and non-ASD group. There was no significance in the test for heterogeneity and the studies had low heterogeneity (two *p* for heterogeneity = 0.04, 0.41; *I*^2^ = 45, 0%, Fig. 13). The meta-analysis showed that preoperative LL was not associated with a significant increase in the incidence of ASD; however, post-operative LL was associated with it (fixed-effects model; *p* = 0.10, 0.002, SMD = −0.75, 95% CI [−1.65, 0.14]; SMD = −3.70, 95% CI[−5.99, −1.42], respectively, Fig. 13).

### Publication bias

After detection of publication bias by STATA 12.0, there was no publication bias found for all included studies (all *p* > 0.05).

## Discussion

Degenerative lumbar diseases are common diseases in the clinic, especially in the elderly population. Posterior lumbar fusion surgery is a popular surgical procedure in treatment for patients with degenerative spinal disorders. ASD, as a complication of posterior lumbar spinal fusion surgery, always attracts the attention of spine surgeons [3, 6, 8, 9]. In 2004, Paul Park [31] reviewed articles involved ASD after lumbar fusion surgery and concluded that age, posterior lumbar interbody fusion, injury to the facet joint of the adjacent segment, long-segment fusion, sagittal alignment, pre-existing degenerated disc at the adjacent level, LL, osteoporosis, female gender, post-menopausal state were potential risk factors for ASD. Furthermore, in 2012, Brandon D [32] performed a mate-analysis on ASD indicating that age more than 60 years, male sex, facet degeneration, degenerative disc disease, adjacent to the fused segment, multilevel fusion, fusion to L5, and excessive disc height distraction. However, this article was limited to higher-quality studies because of only 5 included studies. Although many scholars pay more attention to ASD after spine surgery, the risk factors associated with ASD are controversial.

Thus, we perform a meta-analysis to evaluate the risk factors associated with ASD [12–30]. The rate of ASD after posterior lumbar fusion surgery was 18.6% (ranged from 8.5 to 69.4%) in this study. The pooled results from this meta-analysis suggested that gender of patients, history of diabetes, BMD, preoperative ODI and JOA, the type of fusion (PLIF vs TLIF), type of bone graft (auto- vs allograft), fusion to S1 (vs non-fusion to S1), diagnose (lumbar disc herniation, lumbar spinal stenosis, lumbar spondylolisthesis), preoperative PT, LL and SS, post-operative SS, PT, and PI were not were associated with a significant increase in the incidence of ASD. However, older age, BMI, the history of smoking and hypertension, preoperative adjacent disc degeneration, long-segment fusion, superior facet violation, high lumbosacral joint angle, pre- and post-operative L1-S1 SVA, post-operative LL, and preoperative PI were associated with a significant increase in the incidence of ASD.

Aota [33] observed that patients older than 55 years of age were at risk of ASD. However, some articles [12, 16, 19] indicated that age was not a significant factor of ASD. In our study, the older age demonstrated an increased risk of developing ASD. The reason that the older spine is less flexible and more difficult to adapt to the biomechanical alterations after fusion might partially explain this difference [34].

Bagheri [35] demonstrated that patients who had higher preoperative BMI showed a statistically increase in the risk of developing ASD. Wang [15] reported that BMI more than 25 kg/m^2^ was found to be a risk factor for ASD. Our results were consistent with the previous studies [12, 15, 35]. In the present study, the history of smoking and hypertension was considered a risk of ASD, but the reason was unexplained.

Anandjiwala [26] demonstrated that preoperative disc degeneration at an adjacent level was a significant indicator of developing ASD. Our finding confirmed that patients with preoperative Pfirrmann’s classification of more than 3 in the radiographic adjacent segment were found to be a statistic risk factor of ASD. Compared with patients whose Pfirrmann’s classification was less than 3, biomechanical alterations caused by fusion make it more vulnerable to experience degeneration at the adjacent level that preoperative Pfirrmann’s classification was more than 3. Additionally, we also found that preoperative superior facet violation was related to the increasing rate of ASD. Actually, preoperative superior facet violation is a form of degeneration at the adjacent segment, causing deduced adaptability to biomechanical change.

Long fusion that was more than 3 levels demonstrated a significant relationship with the incidence of ASD. Ghiselli et al. [36] reported that multiple-level fusion had a three times higher risk for developing ASD than single-level fusion. Decreased elasticity and increased stiffness of the lumbar segment caused by long fusion are difficult to accommodate biomechanical changes at the adjacent motion segment including stress concentration and intradiscal pressures, which make it easier to experience degeneration at the adjacent segment.

It remains controversial as to whether an association exists between sagittal malalignment and ASD. Anandjiwala [26] showed that sagittal alignment parameters were not associated with the development of ASD. Zhong [19] demonstrated the same results. While other articles showed that patients with post-operative sagittal imbalance have a statistically significant increased chance of developing ASD [37, 38], our finding showed that partial sagittal parameters had a close relationship with the development of ASD. Djurasovic [39] found the patients developing ASD with a significantly lower level of LL. Wu [40] reported that the post-operative angle of LL was 7.9° higher than the preoperative angle in patients after PLIF. Nakashima [41] concluded that appropriate post-operative LL after surgery could play a crucial role in the prevention of ASD. In the present study, post-operative LL was found to be a risk of ASD, while preoperative LL was not. Nakashima [42] believed that the patients with a high preoperative PI value have a significantly higher risk of ASD after spinal fusion, but he did not explore whether post-operative PI is the risk of ASD. In our study, regarding the role of preoperative PI to ASD, we were consistent with Nakashima. Nevertheless, post-operative PI was not associated with a significant increase in the incidence of ASD.

Kumar [43] showed that patients with a normal C7 plumb line alignment had a lower incidence of adjacent-level change following lumbar spinal fusion. Liang [16] found that patients with normal post-operative lumbar sagittal alignment had a lower incidence of ASD. In addition, he concluded that the preoperative L1-S1 SVA was found to be a potential risk factor for predicting ASD after lumbar spine fusion. Our findings showed that both preoperative and post-operative L1-S1 SVA were associated with a significant increase in the incidence of ASD. Correction of sagittal alignment by spinal fusion plays an important role in the development of ASD. It ensures the proper conditions for fusion and facilitates the preservation of the adjacent segment. Abnormal sagittal alignment can have a deleterious effect on the adjacent segment.

There were several limitations to this study. First, we evaluated only radiological and asymptomatic ASD, but symptomatic one was not considered. Additionally, some factors had two included studies. The mentioned above might impact the accuracy of the results. Second, some factors, like PI-LL, might be risk factors for ASD. Because related studies were few and could not get pooled result, we excluded them. Third, follow-up time varied between the studies and thus may influence our results. Fourth, all the included studies come from Asian countries, which may affect the bias of results.

In conclusion, older age, BMI, the history of smoking and hypertension, preoperative adjacent disc degeneration, long-segment fusion, superior facet violation, high lumbosacral joint angle, pre- and post-operative L1-S1 SVA, post-operative LL, and preoperative PI were associated with a significant increase in the incidence of ASD. In this meta-analysis, we can clearly see which kind of people more likely had ASD after surgery. We hope this article can provide a reference for spinal surgeons in the treatment of lumbar degeneration diseases. Meanwhile, it is helpful for future study on ASD. Further large-scale, well-designed studies are urgently needed.

## Supplementary Information

**Additional file 1.**

## Data Availability

Yes.

## Abbreviations

ASD: Adjacent segment degeneration
BMI: Body mass index
BMD: Bone mineral density
LL: Lumbar lordosis
PI: Pelvic incidence
PT: Pelvic tilt
SS: Sacral slope
SVA: Sagittal vertical axis
