# Supplementary material for: Risk factors for adjacent segment degeneration after posterior lumbar fusion surgery in treatment for degenerative lumbar disorders: a meta-analysis
Source: J Orthop Surg Res. 2020 Dec 3;15:582. doi: 10.1186/s13018-020-02032-7 (PMC7713357; doi:10.1186/s13018-020-02032-7)
Supplement: Supplementary file 1 — Additional file 1. [file 13018_2020_2032_MOESM1_ESM.docx]

**Reference:**

1.G. Cheh, K. H. Bridwell, L. G. Lenke et al. “Adjacent segment disease followinglumbar/thoracolumbar fusion with pedicle screw instrumentation: a minimum 5-year follow-up,” Spine, vol. 32, no. 20, pp. 2253–2257, 2007.

2.Greenwood J, McGregor A, Jones F, et al. Rehabilitation followinglumbar fusion surgery: a systematic review and meta-analysis. Spine(Phila Pa 1976) 2016;41:E28–36.

3.Lee CS, Hwang CJ, Lee SW et al (2009) Risk factors for adjacent segment disease after lumbar fusion. Eur Spine J 11:1637–1643

4.Quinell RC, Stockdale HR (1981) Some experimental observations on the influence of a single floating fusion on the remaining lumbar spine. Spine 6:263–267.

5.Battie MG, Videman T, Parent E (2004) Lumbar disc degeneration: epidemiology and genetics influences. Spine 29:2679–2690

6. Puvanesarajah V, Cancienne JM, Werner BC, et al. Perioperative complications associated with posterolateral spine fusions: a study of elderly medicare beneficiaries. The Spine Journal 2016;16: S266-7 [Epub ahead of print].

7. Park P, GartonHJ, Gala VC, et al. Adjacent segment disease after lumbar or lumbosacral fusion: review of the literature. Spine (Phila Pa 1976) 2004;29:1938–44.

8. Kumar MN, Baklanov A, Chopin D. Correlation between sagittal plane changes and adjacent segment degeneration following lumbar spine fusion. Eur Spine J 2001;10:314–9.

9. Etebar S, Cahill DW (1999) Risk factors for adjacent-segment failure following lumbar fixation with rigid instrumentation for degenerative instability. J Neurosurg 90:163–169.

10. Ha KY, Chang CH, Kim KW, Kim YS, Na KH, Lee JS (2005) Expression of estrogen receptor of the facet joints in degenerative spondylolisthesis. Spine (Phila Pa 1976) 30:562–566. doi: 00007632-200503010-00015[pii]

11. Xiao SW, Jiang H, Yang LJ, Xiao ZM (2015) Anterior cervical discectomy versus corpectomy for multilevel cervical spondylotic myelopathy: a meta-analysis. Eur Spine J: Off Publ Eur Spine Soc Eur Spinal Deform Soc Eur Sect Cerv Spine Res Soc 24:31–39. doi:10.1007/s00586-014-3607-1.

12.Zheng G, Wang C, Wang T, et al. Relationship between postoperative lordosis distribution index and adjacent segment disease following L4-S1 posterior lumbar interbody fusion. *J Orthop Surg Res*. 2020;15(1):129. doi:10.1186/s13018-020-01630-9

13. Huang Lin, Cai Zhaopeng, Chen Keng, et al. Retrospective analysis of preoperative risk factors of adjacent segment degeneration after posterior lumbar interbody fusion. J Clin Orthop Res. 2017;2(1):1-5.

14.Huang Mi, Yu Miao, Liu Xiaoguang et al. Correlative factors for adjacent segment degeneration after lumbar spinal fusion. Chinese Journal of Spine and Spinal Cord. 2014;24(3):199-203.

15.Wang H, Ma L, Yang D, et al. Incidence and risk factors of adjacent segment disease following posterior decompression and instrumented fusion for degenerative lumbar disorders. *Medicine (Baltimore)*. 2017;96(5):e6032. doi:10.1097/MD.0000000000006032

16.Liang J, Dong Y, Zhao H. Risk factors for predicting symptomatic adjacent segment degeneration requiring surgery in patients after posterior lumbar fusion. *J Orthop Surg Res*. 2014;9:97. doi:10.1186/s13018-014-0097-0

17.Makino T, Honda H, Fujiwara H, Yoshikawa H, Yonenobu K, Kaito T. Low incidence of adjacent segment disease after posterior lumbar interbody fusion with minimum disc distraction: A preliminary report. *Medicine (Baltimore)*. 2018;97(2):e9631. doi:10.1097/MD.0000000000009631

18. Heo Y,  Park JH,  Seong HY et al. Symptomatic adjacent segment degeneration at the L3–4 level after fusion surgery at the L4–5 level: evaluation of the risk factors and 10-year incidence. Eur Spine J 2015;24(11).DOI：[10.1007/s00586-015-4188-3](https://doi.org/10.1007/s00586-015-4188-3)

19. Zhong ZM,  Deviren V,  Tay B et al. Adjacent segment disease after instrumented fusion for adult lumbarspondylolisthesis: Incidence and risk factors. Clin Neurol Neurosurg. 2017; 156 (2017) 29–34 DOI：[10.1016/j.clineuro.2017.02.020](https://doi.org/10.1016/j.clineuro.2017.02.020)

20.Ma Z, Huang S, Sun J, Li F, Sun J, Pi G. Risk factors for upper adjacent segment degeneration after multi-level posterior lumbar spinal fusion surgery. *J Orthop Surg Res*. 2019;14(1):89. doi:10.1186/s13018-019-1126-9

21.Ushio S, Hirai T, Yoshii T, et al. Preoperative Risk Factors for Adjacent Segment Degeneration after Two-Level Floating Posterior Fusion at L3-L5. *Spine Surg Relat Res*. 2019;4(1):43‐49. doi:10.22603/ssrr.2019-0003

22. Seyed Reza Bagheri1, Ehsan Alimohammadi2, Alireza Zamani Froushani et al.Adjacent segment disease after posterior lumbar instrumentation surgery fordegenerative disease: Incidenceand risk factors. Journal of Orthopaedic Surgery.2019;27(2) 1–6

23. Jun Seok Bae, Sang-Ho Lee, Jin-Sung Kim,et al. Adjacent Segment Degeneration After Lumbar Interbody Fusion With Percutaneous PedicleScrew Fixation for Adult Low-Grade IsthmicSpondylolisthesis: Minimum 3 Years of Follow-up.Neurosurgery. 2010; 67:1600–1608, DOI: 10.1227/NEU.0b013e3181f91697

24. Kyoung-Suok Cho,Suk-Gu Kang,Do-Sung Yoo et al.Risk Factors and Surgical Treatment for Symptomatic Adjacent Segment Degenerationafter Lumbar Spine Fusion. J Korean Neurosurg Soc.2009; 46: 425-430,

25.  Jaewan Soh, Jae Chul Lee, Byung Joon Shin. Analysis of Risk Factors for Adjacent Segment Degeneration Occurring More than 5 Years after Fusion with Pedicle Screw Fixation for Degenerative Lumbar Spine. Asian Spine J. 2013;7(4):273-281

26. Jigar Anandjiwala, Jun-Yeong Seo, Kee-Yong Ha et al.Adjacent segment degeneration after instrumented posterolateral lumbar fusion: a prospective cohort study with a minimum five-year follow-up. Eur Spine J. (2011) 20:1951–1960.DOI 10.1007/s00586-011-1917-0

27.  Masayuki Miyagi, Osamu Ikeda, Seiji Ohtori et al. Additional decompression at adjacent segments leads to adjacent segment degeneration after PLIF. Eur Spine J. (2013) 22:1877–1883.DOI 10.1007/s00586-013-2694-8

28. Bai-Ling Chen, Fu-Xin Wei, Kazumasa Ueyama et al. Adjacent segment degeneration after single-segment PLIF: the risk factor for degeneration and its impact on clinical outcomes. Eur Spine J (2011) 20:1946–1950 DOI 10.1007/s00586-011-1888-1

29. Li Weishi, Sun Zhouran, Guo Yang et al. Effect of spinopelvic sagittal alignment on the development of adjacent segment degeneration after posterior lumbar fusion: investigation on cases with a minimum of 6 years of follow up. Chinese Journal of Spine and Spinal Cord.2018;28(10):865-872.

30. Guo Yang, Sun Zhouran, Zhou Siyu, et al. The effect of pre-existing degeneration at adjacent segment on postoperative adjacent segment degeneration and surgical clinical outcome. Spine and Spinal Cord.2020;30(2):103-110.

31. Paul Park, Hugh J. Garton, et al. Adjacent Segment Disease after Lumbar or Lumbosacral Fusion: Review of the Literature. SPINE. 2004. 29(17): pp 1938–1944

32. Brandon D, Lawrence, Jeff Wang et al. Predicting the Risk of Adjacent Segment Pathology After Lumbar Fusion. SPINE. 2012;37(22S): pp S123–S132.

33. Aota Y, Kumano K, Hirabayashi S. Postfusion instability at the adjacent segments after rigid pedicle screw fixation for degenerative lumbar spinal disorders. J Spinal Disord. 1995;8:464–73.

34. Kim YJ, Bridwell KH, Lenke LG, Glattes CR, Rhim S, Cheh G (2008) Proximal junctional kyphosis in adult spinal deformity after segmental posterior spinal instrumentation and fusion: minimum five-year follow-up. Spine 33(20):2179–2184. doi:10. 1097/BRS.0b013e31817c0428

35. Bagheri SR, Alimohammadi E, Zamani Froushani A, et al. Adjacent segment disease after posterior lumbar instrumentation surgery for degenerative disease: Incidence and risk factors. J Orthop Surg (Hong Kong) 2019;27: 2309499019842378.

36. Ghiselli G, Wang JC, Bhatia NN, Hsu WK, Dawson EG (2004) Adjacent segment degeneration in the lumbar spine. J Bone Joint Surg Am 86-A:1497–1503.

37.Zencica P, Chaloupka R, Hladikova J, et al. Adjacent segment degeneration after lumbosacral fusion in spondylolisthesis: a retrospective radiological and clinical analysis. Acta Chir Orthop Traumatol Cech 2010; 77: 124–130.

38. Chung KJ, Suh SW, Swapnil K, et al. Facet joint violation during pedicle screw insertion: a cadaveric study of the adult lumbosacral spine comparing the two pedicle screw insertion techniques. Int Orthop 2007; 31: 653–656.

39. Nakashima H, Kawakami N, Tsuji T, et al. Adjacent segment disease afterposterior lumbar interbody fusion: based on cases with a minimum of 10years of follow-up. Spine (Phila Pa 1976). 2015;40:E831–41.

40. Wu CH, Wong CB, Chen LH, et al. Instrumented posterior lumbar interbodyfusion for patients with degenerative lumbar scoliosis. J Spinal Disord Tech.2008;21:310–5.

41. Djurasovic MO, Carreon LY, Glassman SD, et al. Sagittal alignment as a riskfactor for adjacent level degeneration: a case-control study. Orthopedics.2008;31:546.

42. Nakashima H, Kawakami N, Tsuji T, et al. Adjacent segment disease afterposterior lumbar interbody fusion: based on cases with a minimum of 10years of follow-up. Spine. 2015;40(14):E831–41.

43.Kumar MN, Baklanov A, Chopin D: Correlation between sagittal planechanges and adjacent segment degeneration following lumbar spinefusion. Eur Spine J 2001, 10(4):314–319.

Fig. 1 Flow diagram of studyselection

Fig.2 a. The standardized mean difference (SMD) estimate preoperative age in 2 groups. b. The standardized mean difference (SMD) estimate preoperative body mass index in 2 groups. c. The odds ratio (OR) estimate for history of smoking. CI=confidence interval, df=degrees of freedom, M-H=Mantel–Haenszel.

Fig.3 a. The odds ratio (OR) estimate for gender. b. The odds ratio (OR) estimate for history of diabetes. CI=confidence interval, df=degrees of freedom, M-H=Mantel–Haenszel.

Fig.4 a. The standardized mean difference (SMD) estimate bone mineral density in 2 groups. b. The standardized mean difference (SMD) estimate preoperative ODI score in 2 groups. c. The standardized mean difference (SMD) estimate preoperative JOA score in 2 groups.df=degrees of freedom, ODI=Oswestry disability index. JOA= Japanese Orthopedic Association, M-H=Mantel–Haenszel.

Fig.5 a. The odds ratio (OR) estimate for history of hypertension. b. The odds ratio (OR) estimate for preoperative Pfirrmann’s classification. c. The odds ratio (OR) estimate for length of fusion (short vs long fusion). CI=confidence interval, df=degrees of freedom, M-H=Mantel–Haenszel.

Fig.6 a. The odds ratio (OR) estimate for preoperative superior facet violation. b. The standardized mean difference (SMD) estimate preoperative preoperative lumbarsacral joint angle in 2 groups. CI=confidence interval, df=degrees of freedom, M-H=Mantel–Haenszel.

Fig.7 a. The odds ratio (OR) estimate for type of fusion (PLIF vs TLIP). b. The odds ratio (OR) estimate for type of graft (auto-vs allo-graft). c. The odds ratio (OR) estimate for fusion to S1 (vs non-fusion to S1).CI=confidence interval, df=degrees of freedom, M-H=Mantel–Haenszel.

Fig.8 a. The odds ratio (OR) estimate for diagnosis (lumbar spinal stenosis vs lumbar spondylolisthesis). b. The odds ratio (OR) estimate for diagnosis (lumbar disc herniation vs lumbar spinal stenosis). c. The odds ratio (OR) estimate for diagnosis (lumbar disc herniation vs lumbar spondylolisthesis). CI=confidence interval, df=degrees of freedom, M-H=Mantel–Haenszel.

Fig.9 a. The standardized mean difference (SMD) estimate preoperative L1-S1sagittal vertical axis (SVA)in 2 groups. b. The standardized mean difference (SMD) estimate postoperative L1-S1SVA in 2 groups. CI=confidence interval, df=degrees of freedom, M-H=Mantel–Haenszel.

Fig.10 a. The standardized mean difference (SMD) estimate preoperative pelvic tilt (PT)in 2 groups. b. The standardized mean difference (SMD) estimate postoperative PT in 2 groups. CI=confidence interval, df=degrees of freedom, M-H=Mantel–Haenszel.

Fig.11 a. The standardized mean difference (SMD) estimate preoperative sacral slope (SS) in 2 groups. b. The standardized mean difference (SMD) estimate postoperative SS in 2 groups. CI=confidence interval, df=degrees of freedom, M-H=Mantel–Haenszel.

Fig.12 a. The standardized mean difference (SMD) estimate preoperative pelvic incidence (PI) in 2 groups. b. The standardized mean difference (SMD) estimate postoperative PI in 2 groups. CI=confidence interval, df=degrees of freedom, M-H=Mantel–Haenszel.

Fig.13 a. The standardized mean difference (SMD) estimate preoperative lumbar lordosis (LL) in 2 groups. b. The standardized mean difference (SMD) estimate postoperative LL in 2 groups. CI=confidence interval, df=degrees of freedom, M-H=Mantel–Haenszel.
